# Mapping forest and site quality of planted Chinese fir forest using sentinel images

**DOI:** 10.3389/fpls.2022.949598

**Published:** 2022-10-04

**Authors:** Chongjian Tang, Zilin Ye, Jiangping Long, Zhaohua Liu, Tingchen Zhang, Xiaodong Xu, Hui Lin

**Affiliations:** ^1^ Research Center of Forestry Remote Sensing & Information Engineering, Central South University of Forestry and Technology, Changsha, China; ^2^ Key Laboratory of Forestry Remote Sensing Based Big Data & Ecological Security for Hunan Province, Changsha, China; ^3^ Key Laboratory of State Forestry Administration on Forest Resources Management and Monitoring in Southern Area, Changsha, China

**Keywords:** planted Chinese fir forest, forest quality, site quality, machine learning, sentinel

## Abstract

Normally, forest quality (FQ) and site quality (SQ) play an important role in evaluating actual and potential forest productivity. Traditionally, these assessment indices (FQ and SQ) are mainly based on forest parameters extracted from ground measurement (forest height, age, density, forest stem volume (FSV), and DBH), which is labor-intensive and difficult to access in certain remote forest areas. Recently, remote sensing images combined with a small number of samples were gradually applied to map forest parameters because of the various advantages of remote sensing technology, such as low cost, spatial coverage, and high efficiency. However, FQ and SQ related to forest parameters are rarely estimated using remote sensing images and machine learning models. In this study, the Sentinel images and ground samples of planted Chinese fir forest located in the ecological “green-core” area of Changzhutan urban cluster, were initially employed to explore the feasibility of mapping the FQ and SQ. And then, four types of alternative variables (backscattering coefficients (VV and VH), multi-spectral bands, vegetation indices, and texture characteristics) were extracted from Sentinel-1A and Sentinel-2A images, respectively. After selecting variables using a stepwise regression model, three machine learning models (SVR, RF, and KNN) were employed to estimate various forest parameters. Finally, the FQ of the study region was directly mapped by the weights sum of related factors extracted by the factor analysis method, and the SQ was also extracted using mapped forest height and age. The results illustrated that the accuracy of estimated forest parameters (DBH, H, and Age) was significantly higher than FSV, FCC, and Age and the largest and smallest rRMSEs were observed from FSV (0.38~0.40) and forest height (0.20~0.21), respectively. Using mapped forest parameters, it also resulted that the rRMSEs of estimated FQ and SQ were 0.19 and 0.15, respectively. Furthermore, after normalization and grading, the grades of forest quality were mainly concentrated in grades I, II, and III in the study region. Though the accuracy of mapping FQ and SQ is limited by the saturation phenomenon, it is significantly proved that using machine learning models and Sentinel images has great potential to indirectly map FQ and SQ.

## 1 Introduction

With the aggravation of global warming, forests are regarded as the most critical ecological system on land, are playing an important role in reducing carbon dioxide concentration (Jin et al., 2011; Kavats et al., 2020) and forest productivity is essential to accurately evaluate forest resources. Normally, forest quality (FQ) and site quality (SQ) are considered as important indices in the evaluation of forest productivity, whereby forest managers can assess potential forest stem volume production for a species or forest type (Gong et al., 2007; Fujiki et al., 2016). Traditionally, forest and site quality assessments are mainly based on forest parameters (forest height, age, density, forest stem volume, and DBH) investigated in the field, which is labor-intensive and difficult to access in certain remote forest areas (Venkatalaxmi et al., 2004; Belgiu and Drăguţ, 2016; Che et al., 2018). Therefore, it is urgent to improve the assessment method of forest and site quality for large forest regions.

Traditionally, FQ and SQ were received from the evaluating models constructed by measured or estimated forest parameters. In previous studies, several approaches were employed to construct the models between forest quality and forest parameters in various forest types. Normally, three types of factors, forest productivity, forest structure factors, and topographic factors, are highly related to forest quality (Jugran et al., 2005). Firstly, forest productivity includes indicators that affect the metabolic strength of forest, such as tree height, DBH, and FSV (Reich, 2012). And then, forest structure factors reflect both vertical and horizontal information about the forest, such as forest crown closure (FCC) and density. Topographic factors are also the necessary information to describe the slope position and the orientations. Furthermore, the analytic hierarchy process (AHP) has been used to determine the weights of related factors, and then the values of FQ were eventually calculated by weighted average (Shataee et al., 2012; Zhang et al., 2015; Feng et al., 2016). However, it is still a problem to determine the contribution of each related factor. As for SQ, tree height is usually used as an evaluation index derived from national forest inventory data (Yu et al., 2019). There are two traditional methods to evaluate SQ, the site class method and the site index method (Lei et al., 2018). The site index method was formed using the relationship between dominant tree height and forest age, which has a complete theoretical system (Lumbres et al., 2018). However, extracting forest parameters severely hinders the mapping of the FQ and SQ in large regions.

Over the last few decades, remote sensing (RS) is becoming an increasingly important technology in mapping forest parameters. Normally, forest parameters, such as forest height, age, density, FCC, DBH, FSV, etc., have been widely mapped by various regression algorithms based on multi-spatial resolutions and multi-spectral sensors. Especially, the mean forest heights were estimated using a combination of sentinel 1/2 and DEM using optical and SAR data (Kahriman et al., 2014). And FCC was also mapped from Landsat images (Yu et al., 2017a). Furthermore, FSV was widely estimated using various images acquired from several sensors (Chen et al., 2017). The previous studies indicated that the indirect measurements of forest parameters by utilizing remotely sensed data, have great potential to provide estimated forest parameters more reliable and cost-effective than direct field-based measurements (Wang et al., 2018). However, these complicated forest assessment indies (FQ and SQ) related to forest parameters are rarely estimated using remote sensing images. Therefore, it is meaningful to further processing for mapping FQ and SQ using remote sensing images in region scales.

Facing the challenge of mapping FQ and SQ, the accuracy is depended on models between the forest parameters and variables extracted from images (Zhang et al., 2018). In previous studies, traditional linear regression models were often used to estimate forest parameters (Zhou et al., 2013; Pu and Cheng, 2015). However, these linear models were limited to describing the relationships for these complex nonlinear problems. Therefore, the prediction of forest parameters using machine learning algorithms is a relatively reasonable selection. Recently, prevalent methods, such as k-nearest neighbor (KNN), artificial neural network (ANN), random forest (RF), and support vector regression (SVR), have been widely used in forest parameter estimation (Shao and Lunetta, 2012; Verrelst et al., 2012; Rodriguez-Galiano et al., 2015; Xu et al., 2018) and the resulted showed that machine learning algorithms have more advantages than traditional regression algorithms (Cooner et al., 2016).

Therefore, this paper mainly focused on mapping FQ and SQ using machine learning algorithms and variables extracted forest parameters from Sentinel-1A and Sentinel-2A in the planted Chinese fir forest. Firstly, the values of FQ and SQ in each ground sample were calculated based on the Factor analysis method and forest height-age model, respectively. And then, four types of alternative variables, including backscattering coefficients (VV and VH), multi-spectral bands, vegetation indices and texture characteristics, were extracted from Sentinel -1A and Sentinel -2A images, respectively. Finally, the results of FQ and SQ were estimated using estimated forest parameters by three machine learning models (SVR, RF, KNN) and applied to further clarify the potential capability of mapping FQ and SQ using remote sensing images.

## 2 Material and methods

### 2.1 Study area

**Figure 1:**
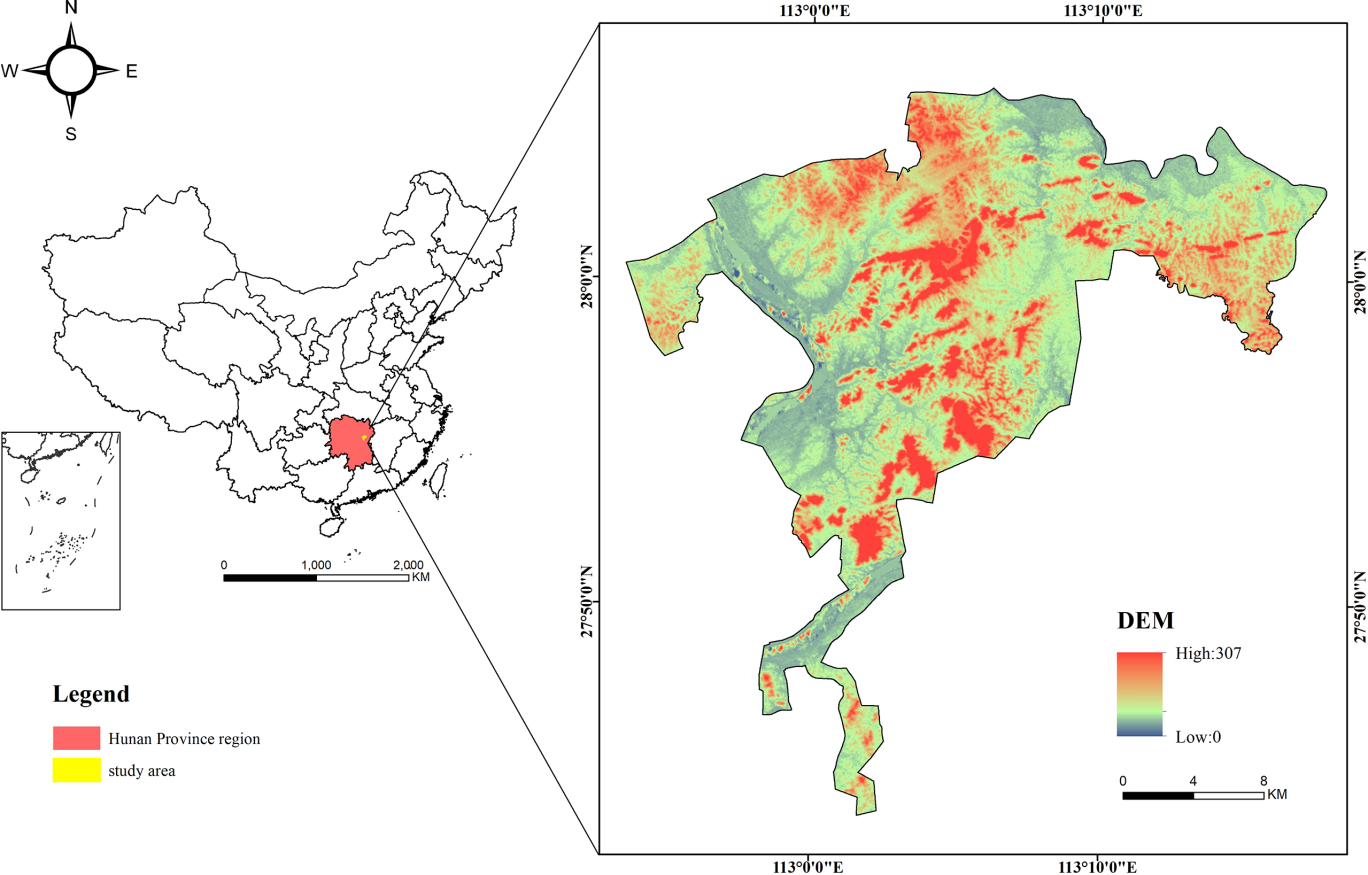
The location of the study area.

The study area is located in the ecological “green-core” area of Changzhutan urban cluster, Hunan Province, China. This area is the intersection of three cities, such as Changsha city, Zhuzhou city, and Xiangtan city, ranging from 112°53′31″E to113°17′47″E and 27°43′29″N to 28°5′53″N (**Figure 1**). There are numerous hills with elevations varying from 15 m to 307 m. Influenced by the subtropical monsoon climate, this region has annual mean and maximum temperatures of 17.09°C and 40°C, respectively. By 2019, forest area and forest biomass are up to 11772 ha and 1.346 million tons, respectively. Moreover, planted Chinese fir is the main tree species in this region, accounting for 68.70% of forest area.

### 2.2 Field data and remote sensing images

**Figure 2:**
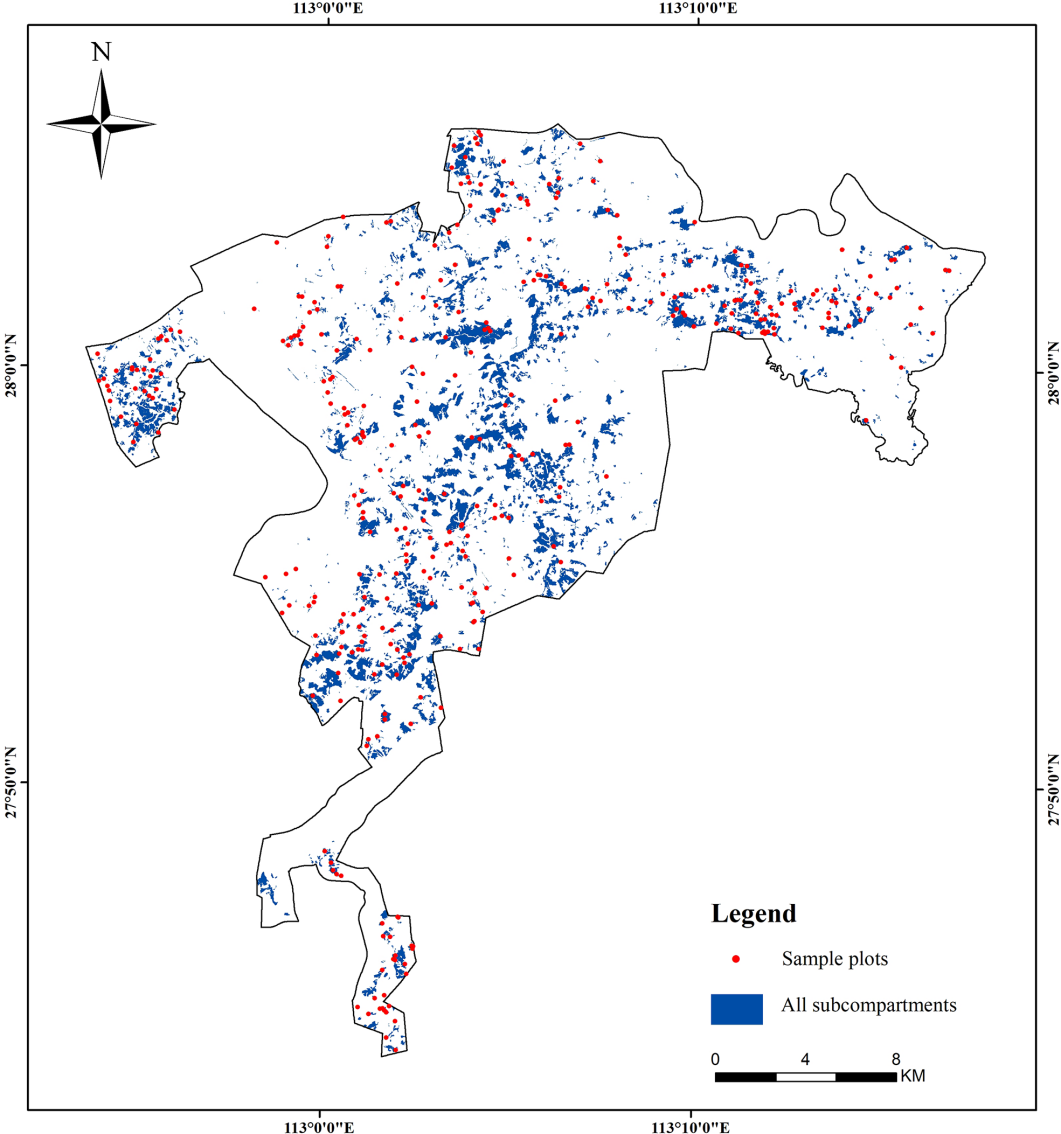
The distribution of the ground samples in the study area.

In the study region, a total of 3,741 sub-compartments were investigated in 2019 and the planted Chinese fir is the dominant tree species (**Figure 2**). In each sub-compartment, forest parameters and geographical factors, such as DBH, H, FCC, FSV, Age, slope, aspect, and slope position, were recorded and established in the database of forest management investigation. According to the stratified random sampling method and the spatial distribution of FQ in planted Chinese fir forest, ten percent of sub-compartments with pure Chinese fir forest were selected from each grade of forest quality. So, 374 sub-compartments (**Figure 2**) were selected as samples to construct the models in the next work. And the statistics results of selected samples are listed in **Table 1**.

**Table 1:** The statistics results of selected samples.

Parameter	Range	Mean	Standard deviation	Coefficient of variation
DBH (cm)	6-24	12.06	3.14	0.26
FSV(m^3^/ha)	8.61-155.56	64.14	32.46	0.51
H(m)	3-14	8.49	2.04	0.24
FCC	0.2-0.9	0.53	0.13	0.25
Density (per ha)	203-2829	1081.30	438.15	0.41
Age(year)	5-30	15.69	5.16	0.33

### 2.3 Pre-processing of remote sensing data

To map the FQ and SQ, two remote sensing images were acquired from Sentinel-1A and Sentinel-2A (**Table 2**), respectively (https://scihub.esa.int). For the images of Sentinel-1A, dual-polarization SAR images with C band (VH and VV) were acquired on September 10, 2019. And then, several pre-processing, including radiometric correction, speckle noise filter, terrain correction, and geocoding, were employed to retrieve the backward scattering coefficient by SNAP software provided by ESA. The multi-spectral images with three spatial resolutions (10m, 20m, and 60m) were acquired on September 5, 2019, and radiation correction, geometric correction, and atmospheric correction were applied to reduce the errors caused by the influence of interference factors. In our study, ten bands with spatial resolutions of 10m and 20m were selected to extract remote sensing variables. Furthermore, the DEM data was derived from the SRTM 30m Digital Elevation Data Product downloaded from the geoscience data cloud (http://www.Gscloud.cn//).

**Table 2:** The information of acquired remote sensing data.

Sensors	Acquisition date	Spectral/Polarizations
Sentinel-1A(level-GRD)	July 10, 2019	VH, VV
Sentinel-2A (level-L2A)	September 10, 2019	Band2, Band3, Band4, Band5, Band6, Band7, Band8, Band8A, Band11, Band12

### 2.4 Variable selection and models

#### 2.4.1 Extracting variables

**Figure 3:**
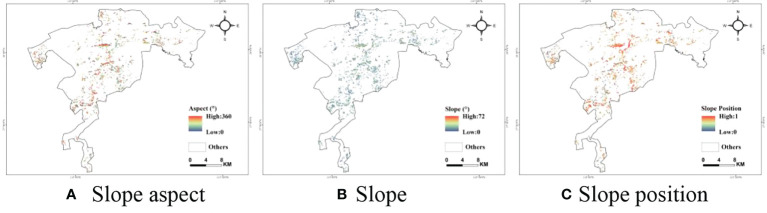
Maps of the topographic factors derived from DEM. **(A)** Slope aspect **(B)** Slope **(C)** Slope position.

After pre-processing, four types of variables, including backscattering coefficients (VV and VH), multi-spectral bands, vegetation indices, and texture characteristics, were extracted from Sentinel -1A and Sentinel -2A images, respectively. In particular, the multi-spectral bands included Band 2, Band 3, Band 4, Band 5, Band 6, Band 7, Band 8, Band 8A, Band 11, and Band 12. Furthermore, eighteen vegetation indices (VIs) were calculated using these selected multi-spectral bands (**Table 3**). In addition, eight texture features (mean, variance, uniformity, contrast, dissimilarity, entropy, second moment, and correlation) were also calculated from images of backscattering coefficients and multi-spectral bands with a size of 3 × 3 (Haralick et al., 1973; Biesiada and Duch, 2007). Additionally, topographic variables, such as the aspect, slope, and slope position, were directly derived from external DEM data (**Figure 3**).

**Table 3:** Selected VIs used for forest parameter estimation.

Vegetation Indices (VIs)	Formula
Ratio vegetation index (RVI)	B8/B4
Difference vegetation index (DVI)	B8-B4
Weighted difference vegetation index (WDVI)	B8-0.5×B4
Infrared vegetation index (IPVI)	B8/(B8+B4)
Perpendicular vegetation index (PVI)	sin (45°) ×B8-cos (45°)×B4
Normalized difference vegetation index (NDVI)	(B8-B4)/(B8+B4)
NDVI with band4 and band5 (NDVI45)	(B5-B4)/(B5+B4)
NDVI of green band (GNDVI)	(B7-B3)/(B7+B3)
Soil adjusted vegetation index (SAVI)	1.5×(B8-B4)/8×(B8+B4+0.5)
Transformed soil adjusted vegetation index (TSAVI)	0.5×(B8-0.5×B4-0.5)/(0.5×B8+B4-0.15)
Modified soil adjusted vegetation index (MSAVI)	(2-NDVI×WDVI) ×(B8-B4)/8×(B8+B4+1-NDVI×WDVI)
Secondly modified soil adjusted vegetation Index (MSAVI2)	0.5×[2×(B8+1)- $\sqrt{2\times {\left(B8+1\right)}^{2}\text{-8×(B8-B4)}}$
Atmospherically resistant vegetation index (ARVI)	B8-(2×B4-B2)/B8+(2×B4-B2)
Pigment specific simple ratio chlorophyll index (PSSRa)	B7/B4
Meris terrestrial chlorophyll index (MTCI)	(B6-B5)/(B5-B4)
Modified chlorophyll absorption ratio index (MCARI)	[(B5-B4)-0.2×(B5-B3)] ×(B5-B4)
Sentinel-2 red edge position index (S2REP)	705+35×[(B4+B7)/2-B5]/(B6-B5)
Global environmental monitoring index (GEMI)	[2×(B8A-B4) +1.5×B8A+0.5×B4]/(B8A+B4+0.5)

#### 2.4.2 Variables selection and models

To obtain the optimally combined variables, the Random Forest Importance was used to select the variables extracted from the alternative feature set. Initially, the Random Forest Importance between the variables and each related forest parameter was calculated and sorted. The optical variable set related to each forest parameter was ranked by stepwise regression, and then the results were used to estimate forest parameters in the next work.

Normally, the accuracy of estimated forest parameters (DBH, FSV, H, FCC, and Density) is highly related to the employed models. In this study, three machine learning models, such as the support vector machine model (SVR), random forest model (RF), and K-nearest neighbor method (KNN), were employed to estimate the forest parameters (Cortes and Vapnik, 1995; Breiman, 2001; Mountrakis et al., 2011). To evaluate the estimated forest parameters, the leave-one-out cross-validation (LOOCV) was employed to calculate the Root Means Square Error (RMSE) and the coefficient of determination ( *R* ^2^ ) between estimated and observed forest parameters. The relative RMSE (rRMSE) was also considered as an accuracy index. The formulas of these indices are listed as follows:


(1) $$R^{2}=1-\frac{\sum_{i=1}^{\text{n}}{\left({\text{y}}_{\text{i}}-{\hat{y}}_{\text{i}}\right)}^{2}}{\sum_{i=1}^{\text{n}}{\left({\text{y}}_{\text{i}}-\bar{y}\right)}^{2}}\;$$

(2) $$RMSE=\sqrt{\frac{\sum_{i=1}^{\text{n}}{\left({\text{y}}_{\text{i}}-{\hat{y}}_{\text{i}}\right)}^{2}}{n}}$$

(3) $$rRMSE=RMSE/y{\;}^{-}\;\times 100\%$$

In where, $\hat{y}$ and y are the estimated and measured forest parameters, respectively.

### 2.5 Forest quality and site quality

Commonly, FQ is highly related to forest productivity, forest structure factors, and topographic factors, such as DBH, FSV, H, density, etc. However, quantifying the contribution of each factor to forest quality is still a knotty problem. In the previous study, the complex problems were often solved by the factor analysis method and analytic hierarchy process (AHP). In our study, the relative importance of each selected factor was determined by the factor analysis method. And the three indicators (Forest productivity, Forest structure, and Topographic factors) were firstly divided by rotating the loading matrix. Then, the degree of contribution of each indicator and indicator group can be obtained based on their eigenvalues, and the relative and global weights of each factor were ultimately determined (**Table 4**).

**Table 4:** FQ evaluation indicators and weights.

Objective	Indicator groups	Relative weights of indicator groups	Indicators	Relative weights	Global weights
FQ	Forest productivity	0.482	DBH	0.356	0.172
			FSV	0.294	0.142
			H	0.350	0.169
	Forest structure	0.281	FCC	0.504	0.142
			Density	0.496	0.139
	Topographic factors	0.237	Aspect	0.325	0.077
			Slope	0.335	0.079
			Slope position	0.340	0.080

Normally, the growth of timber volume or tree height is positively correlated with the growth potential of the stand, and the tree height can generally reflect the change in timber volume in the same-age forest (Duan et al., 2013). Therefore, estimating average stand height is the most effective technique for evaluating stand quality. In our study, the table of site index of Chinese fir forest in Hunan Province (Liu et al., 1982) was employed to evaluate the stand quality based on empirical equations of forest height and age. After determining the stand quality, the values of SQ were also divided into five grades with an interval of 0.2 (**Table 5**).

**Table 5:** The grades of FQ and SQ.

Grades	Ⅰ	Ⅱ	Ⅲ	Ⅳ	Ⅴ
FQ index	(0.0-0.2]	(0.2-0.4]	(0.4-0.6]	(0.6-0.8]	(0.8-1.0]
SQ index	(0.0-0.2]	(0.2-0.4]	(0.4-0.6]	(0.6-0.8]	(0.8-1.0]

To further analyse the relative levels of FQ and SQ, the normalization method was employed. The normalized formula is as follows:


(4) $$x^{*}=\frac{x-x_{min}}{x_{max}-x_{min}}$$

Where, x*is the normalized FQ or SQ, x_max_ is the maximum value of FQ or SQ, and x_min_ is the minimum value of FQ or SQ. And then, the values of FQ or SQ were divided into five grades with an interval of 0.2 (**Table 5**).

## 3 Results

### 3.1 The results of variable selection

To estimate the various forest parameters, several types of variables (backscattering coefficients, multi-spectral bands, vegetation indices, and texture characteristics) were extracted from Sentinel-1A and Sentinel-2A images. And then, the importance between each variable and forest parameters was evaluated by RF. Finally, the optimal variable set extracted by the stepwise regression model was employed to construct the models (**Table 6**). The results indicated that the importance of the variables (mtci and s2rep) of DBH, H, FCC, and Age are relatively higher than other forest parameters. Moreover, it is also illustrated that the variables extracted from Sentinel-1 could be selected for estimating forest parameters.

**Table 6:** The optimal variables set related to each forest parameter.

DBH	FSV	H	FCC	Density	Age
mtci	B12	mtci	pssra	B2_me	mtci
s2rep	mtci	s2rep	rvi	B7_se	s2rep
B8a_cor	VH	B5	B2	B2	B11
pssra	s2rep	VH	gndvi	VV_var	B5_ent
VH_se	pssra	B6	VV_cor	B8a_cor	VV_cor
B8a_var	B8a_cor	B8a_cor	VH_cor	ndi45	B11_me
VH_ent	B12_cor	B2_var	B11_cor	B8a_var	mcari
B8a_con		B4_var	tsavi	B7_ent	B8a_cor
			B12_cor	mtci	VH_cor
			VH_uni	B8a_con	B5
			VV	VH_cor	VH_me
			VV_var	VH_me	B12_var
			B8a_cor	B2_con	B12_cor
			msavi		VV_var
					B3_se
					B2_me

### 3.2 The results of estimated forest parameters

After the variables selection, three models (SVR, RF, and KNN) were employed to construct the relationships between the measured forest parameters and selected variables. And then, the determination coefficient (R^2^) and relative RMSE (rRMSE) were regarded as accuracy indices to evaluate the estimated forest parameters (**Table 7**). The results illustrated that the values of rRMSE varied with forest parameters and models. The largest and smallest rRMSEs were observed from FSV (0.38~0.40) and H (0.20~0.21), respectively. Obviously, the accuracy of estimated forest parameters (DBH, H, and Age) was significantly higher than FSV, FCC, and Age. Additionally, some models are less effective in estimating parameters, such as FCC using the random forest model.

**Table 7:** The R^2^ and rRMSE of the estimated forest parameters.

Model	DBH		H		FSV		FCC			Density		Age	
	R^2^	rRMSE	R^2^	rRMSE	R^2^	rRMSE	R^2^		rRMSE	R^2^	rRMSE	R^2^	rRMSE
SVR	0.28	0.23	0.34	0.20	0.49	0.40	0.42		0.22	0.49	0.22	0.34	0.27
RF	0.34	0.22	0.32	0.21	0.61	/	/		0.30	0.19	0.27	0.25	0.29
KNN	0.34	0.21	0.35	0.20	0.55	0.38	0.13		0.27	0.41	0.23	0.28	0.28

**Figure 4:**
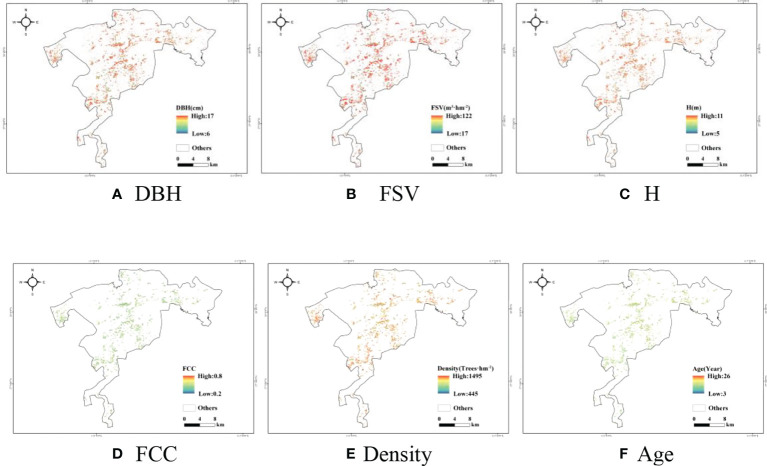
Maps of the forest parameters estimated by optimal models. **(A)** DBH **(B)** FSV **(C)** H **(D)** FCC **(E)** Density **(F)** Age.

Then, the various forest parameters were mapped using the optimal machine learning algorithms according to the results in **Table 7**, and the maps of forest parameters are shown in **Figure 4**. It is illustrated that the ages of planted Chinese fir forests ranged from 6 years to 16 years, and more than 70% of forests’ ages ranged from 13 years to 16 years (**Figure 4F**). It is inferred that most Chinese fir forests are immature forests, and the rest are young forests in the study area. Therefore, most of the forest height (**Figure 4C**) and DBH (**Figure 4A**) ranged from 9 m to 11m and 11cm to 13 cm, respectively. Meanwhile, the FSV is mainly distributed from 70 m^3^/ha to 110 m^3^/ha (**Figure 4B**).

### 3.3 Estimated forest quality and site quality

**Figure 5:**
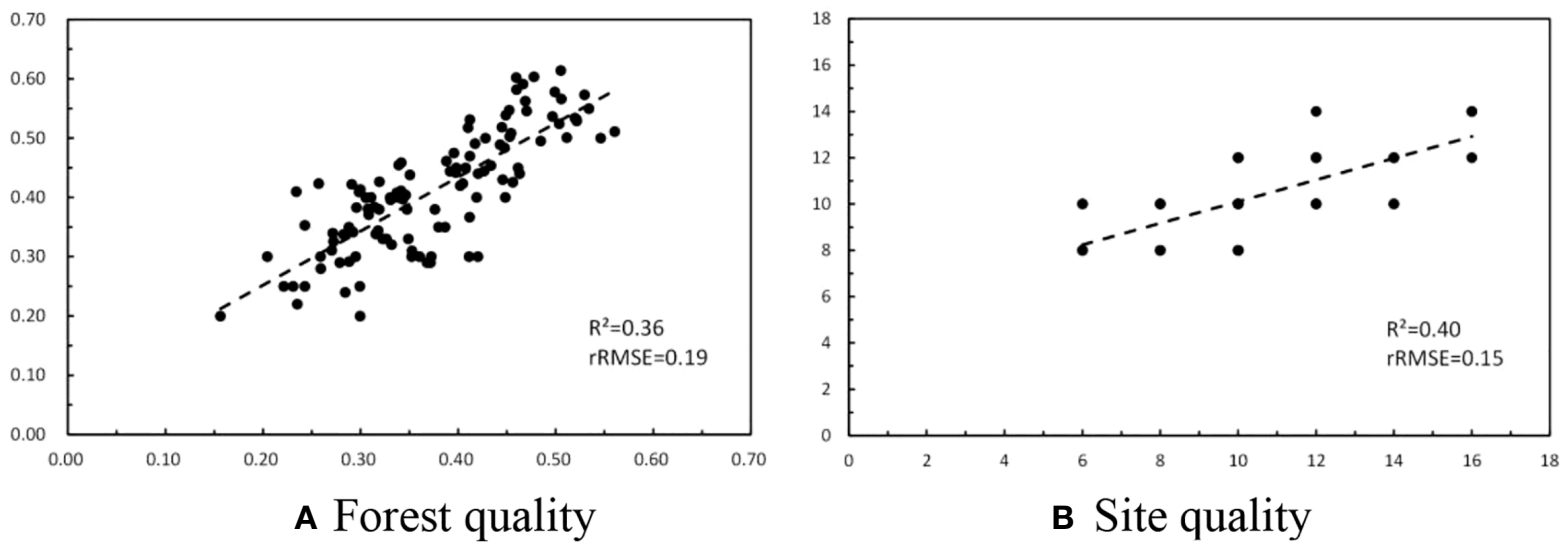
Scatterplots between ground measured and estimated forest quality and site quality. **(A)** Forest quality **(B)** Site quality.

After mapping forest parameters using Sentinel-1A and Sentinel-2A images, the FQ of the study region was directly derived by the weights sum of related factors. Simultaneously, the site quality was also extracted using mapped forest height and ages. Before the normalization, the scatterplots between estimated and measured forest and site quality were illustrated in **Figure 5**. The determination coefficients (R^2^) of the models(Y=X) were 0.36 for forest quality and 0.47 for site quality, respectively. Moreover, the accuracy of estimated forest quality[(rRMSE=0.19)] is lower than site quality (rRMSE=0.15).

**Figure 6:**
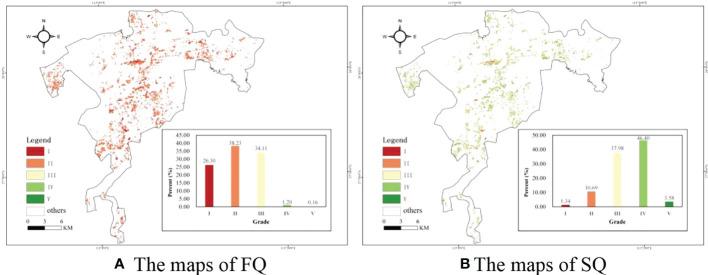
The Maps of the graded FQ and SQ and the histograms of five grades. **(A)** The maps of FQ **(B)** The maps of SQ.

After estimating the FQ and SQ, normalization and grading processes were applied in further analysis of the spatial distribution. Then, five grades (I, II, III, IV, and V) of FQ and SQ were formed, and the spatial distributions of the FQ and SQ were illustrated in **Figure 6**, five grades were represented by five colors (red to green), respectively. The closer to green, the higher the grade, and vice versa. **Figures 6A, B** illustrated that the grades of forest quality were mainly concentrated in grade I, grade II and grade III. Specifically, the percentage of forest quality in grade I was the highest (40.85%), followed by grade II (27.66%) and grade III (23.85%), and the lowest was grade V (1.94%). It is inferred that the forest quality is rather low for planted Chinese fir forests in the study area. Moreover, **Figure 6C** illustrated the distribution of mapped site quality. The percentage of site quality was concentrated in grade III (29.06%) and grade IV (64.6%). However, the sum of grade I and grade II was less than 5%. Therefore, the level of site quality is very high for planted Chinese fir forests in the study area.

### 3.4 The distribution of FQ and SQ

**Figure 7:**
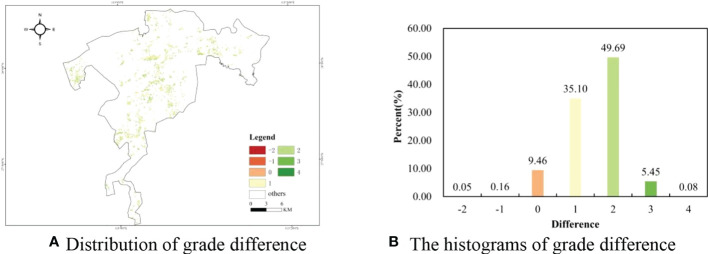
The maps and histograms of difference grades between FQ and SQ. **(A)** Distribution of grade difference **(B)** The histograms of grade difference.

According to the spatial distribution of graded forest FQ and SQ, the matching degree of FQ and SQ is easy to explore in the planted Chinese fir forest. To further analyze the matching degree, the grade difference was calculated by subtracting grades of site quality from grades of forest quality in each sub-compartment. The maps of grade difference were illustrated in **Figure 7A**, and the values contained seven grades ranging from -2 to 4 for the study area (**Figure 7B**). The values ranged from -2 to 0, indicating that the grade of FQ lagged the grade of SQ, and the smaller the grade difference, the worse the mismatch between the FQ and SQ. Furthermore, the values ranged from 0 to 4, meaning that the growth of planted Chinese fir forest broke the limitation of SQ. **Figure 7B** shows the histogram of grades difference between FQ and SQ, and the percentages of values greater than zeros were close to 90% in the planted Chinese fir forest. It was concluded that FQ matched well with SQ in our study area.

## 4 Discussion

### 4.1 The errors of estimated forest parameters

For evaluating FQ and SQ, several forest parameters (DBH, FSV, H, FCC, Density, and Age) are initially estimated using related samples and variables (Silva Guimarães et al., 2020). Therefore, the accuracy of mapped FQ and SQ are highly related to the quality of forest parameters. Moreover, the errors in estimating forest parameters are severely dependent on the employed models and variable sets derived from Sentinel-1A and Sentinel-2A images (Zhao et al., 2019). Therefore, the variables selection methods are a key point in mapping FQ and SQ. Normally, feature selection methods can be classified into three categories: filters, wrappers, and embedded. In our study, four types of alternative variables (backscattering coefficients, multi-spectral bands, vegetation indices, and texture characteristics) were extracted from Sentinel -1A and Sentinel -2A images, respectively (Ghasemi et al., 2018; Gao et al., 2020). Then, two methods (filters and wrappers) of feature selection were applied to obtain the optical variable set, which was ultimately derived by means of a stepwise regression model (Yu et al., 2017a; Yu et al., 2017b; Zhang et al., 2018). It is illustrated that the selected variables set changed with the related forest parameters (**Table 8**), and the errors of estimated forest parameters were related to the selected variables set. For each forest parameter, more than one variable extracted from Sentinel-1A were selected in the optimal variable set, and these features were proved to improve the accuracy of mapping forest parameters (**Table 8**).

**Table 8:** The results of estimated forest parameters using different images data.

Model	Data source	DBH		H		FSV		FCC		Density		Age	
		R^2^	rRMSE	R^2^	rRMSE	R^2^	rRMSE	R^2^	rRMSE	R^2^	rRMSE	R^2^	rRMSE
SVR	S1	0.26	0.21	0.16	0.20	0.53	0.38	0.35	0.23	0.47	0.22	0.33	0.27
	S2	0.13	0.25	0.14	0.23	0.36	0.45	0.20	0.26	0.37	0.24	0.12	0.31
	S1&S2	0.28	0.23	0.34	0.20	0.49	0.40	0.42	0.22	0.49	0.22	0.34	0.27
RF	S1	0.30	0.22	0.25	0.18	0.60	0.35	0.16	0.31	0.19	0.27	0.24	0.29
	S2	0.14	0.25	0.24	0.22	0.44	0.42	0.29	0.24	0.07	0.29	0.05	0.34
	S1&S2	0.34	0.22	0.32	0.21	0.61	0.35	0.12	0.30	0.19	0.27	0.25	0.29
KNN	S1	0.32	0.22	0.18	0.20	0.54	0.38	0.13	0.27	0.43	0.23	0.29	0.28
	S2	0.01	0.26	0.28	0.22	0.37	0.44	0.27	0.24	0.24	0.26	0.08	0.34
	S1&S2	0.34	0.21	0.35	0.20	0.55	0.38	0.13	0.27	0.41	0.23	0.28	0.28

Furthermore, forest parameters (FSV, FCC, and density) are widely estimated using optical satellite images with high spatial resolution, and the values of rRMSE ranged from 15% to 40%. In our study, the rRMSE ranged from 22% to 40%, and the biggest rRMSE was derived from FSV using the SVR model (**Table 8**). Meanwhile, without direct correlation, other forest parameters (DBH, H, and age) are rarely derived from optical satellite images (Wolter et al., 2009; Yalew et al., 2016; Yang et al., 2018). In our study, dual-polarization SAR images with C band (VH and VV) were added to improve the accuracy and reliability in estimating some forest parameters, and the R^2^ ranged from 0.25 to 0.34. Additionally, common machine learning models (SVR, RF, and KNN) are often used to invert these forest parameters. In our study, the accuracy of estimated forest parameters (DBH, H, FCC, and Age) was significantly higher than FSV and density. Especially, the values of rRMSE from estimated DBH, forest height, FCC, and ages were less than 20% using employed models, and those values of FSV and density were larger than 35%. Furthermore, the values of R^2^ ranged from 0.25 to 0.37 for estimated FCC and density because of using remote sensing images with low spatial resolution. Normally, FCC and density were often mapped using high spatial resolution images, even using the images acquired from aerial photography or unmanned aerial vehicles (UAVs) (Wang et al., 2017). Therefore, these estimated forest parameters with low accuracy decreased the reliability of mapping FQ and SQ.

### 4.2 The uncertainty of estimated forest and site quality

**Figure 8:**
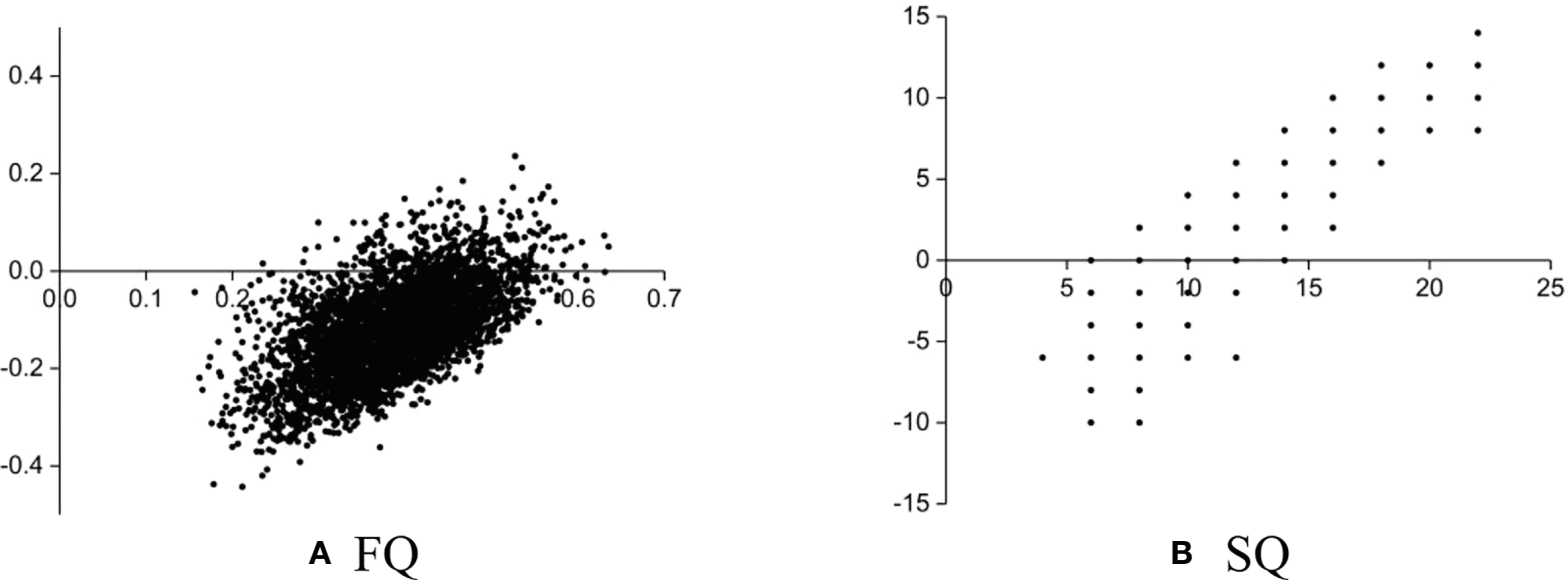
Errors between estimated and measured FQ and SQ. **(A)** FQ **(B)** SQ.

Normally, remote sensing images cannot directly reflect FQ and SQ, and the uncertainty of estimated FQ and SQ depends on the related forest parameters (Lim et al., 2003; Mirik et al., 2013; Wang et al., 2017; Ou et al., 2019). To evaluate the uncertainty of estimated FQ and SQ using Sentinel images, the errors between estimated and measured FQ and SQ of all sub-compartments were illustrated in **Figure 8**. For the results of FQ (**Figure 8A**), the errors ranged from -0.4 to 0 and were mainly concentrated in grade II and the accuracy was severely limited because of these sub-compartments with underestimated FQ. Though SQ is only related to two factors, forest height and age, the errors of estimated and measured SQ were still systematically distributed because of the saturation phenomenon of related factors (Pueschel et al., 2012).

**Figure 9:**
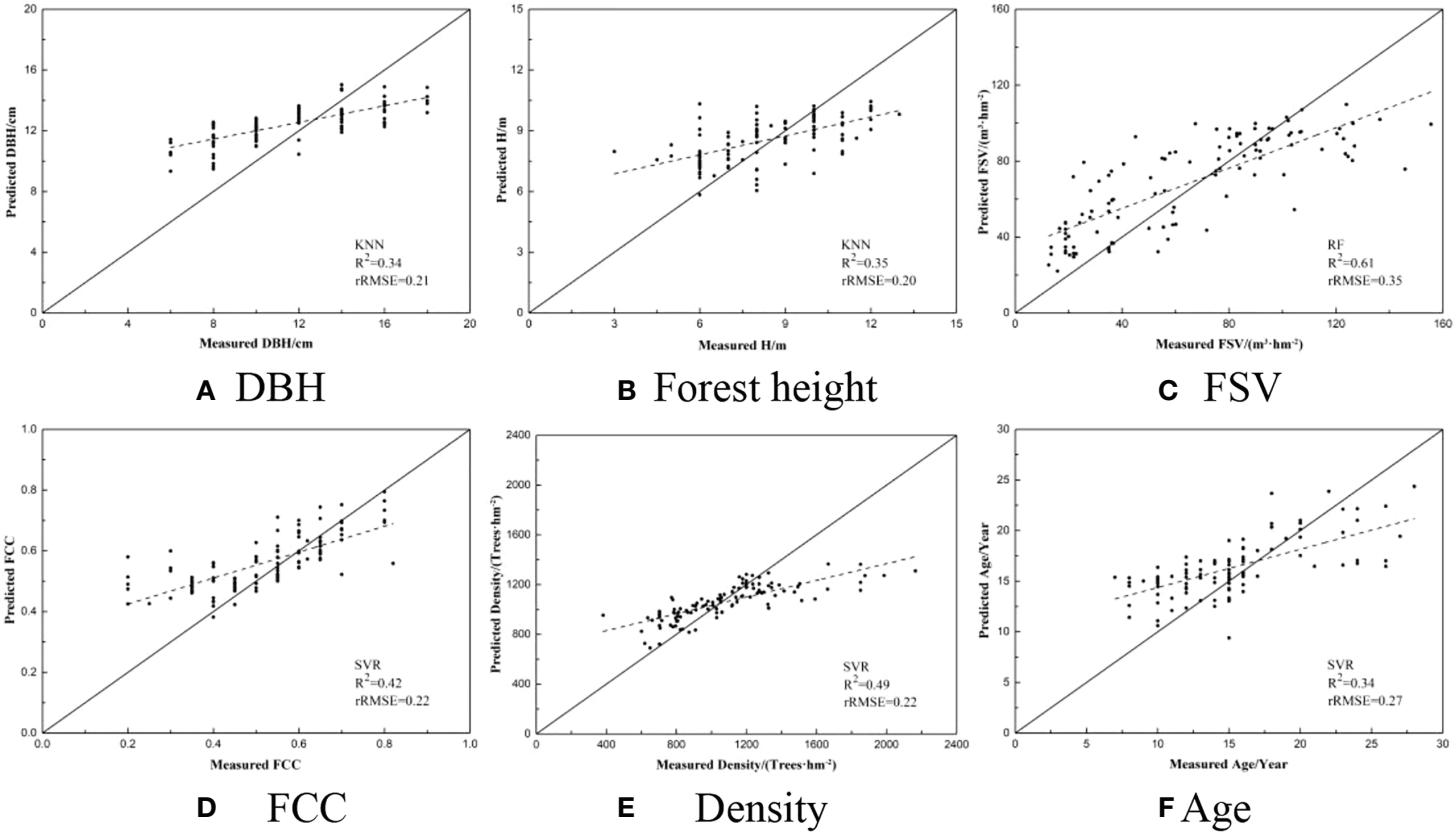
Scatterplots between the ground measured and predicted forest parameters. **(A)** DBH **(B)** Forest height **(C)** FSV **(D)** FCC **(E)** Density **(F)** Age.

In the previous study, for estimating FSV, FCC, or DBH, the saturation phenomenon has been widely proved in many studies (Karlson et al., 2015; Li et al., 2020). It is also found that overestimation may occur in the young forest, while underestimation may be caused by optical saturation (Avitabile and Camia, 2018). Usually, it is reported that the saturation value of FSV often occurs at 200 m^3^/ha for optical images and 300 m^3^/ha for SAR images. In our study, the relationships between estimated and measured forest parameters were illustrated using the estimated results with the highest accuracy of each forest parameter (**Figure 9**). It is obviously found that the saturation phenomenon also occurred for mapping forest height, FSV, density, and age, and the accuracy of these forest parameters was severely affected by underestimated samples. Specially, combined with Sentinel-1A and Sentinel-2A images, the saturation value of FSV closed to less than 120m^3^/ha is significantly lower than other results, and the results are induced by the density and forest ages. In the study area, the average density is larger than 1000 trees/ha and the ages of all sub compartments are around 15 years.

## 5 Conclusions

This study attempts to explore the potentiality of mapping FQ and SQ using Sentinel-1A and Sentinel-2A images. In this study, four types of alternative variables, including backscattering coefficients (VV and VH), multi-spectral bands, vegetation indices, and texture characteristics, were extracted from Sentinel -1A and Sentinel -2A images, respectively. After selecting the optimal variable set using a stepwise regression model, FQ and SQ were indirectly mapped using related forest parameters estimated by three machine learning methods. The results showed that the values of rRMSE of forest and site quality are 0.19 and 0.15, respectively. And the grades of forest quality were mainly concentrated in grades I, II, and III. It has been proved that using related forest parameters has great potential to indirectly estimate forest and site quality. Meanwhile, the results also confirmed that the accuracy of mapped forest and site quality is significantly affected by the estimated errors of forest parameters and the saturation phenomenon. Therefore, further studies will be conducted to delay the saturation phenomenon using high spatial resolution images (such as GF-1 and ZY-3) and polarimetric SAR images.

## Data availability statement

The original contributions presented in the study are included in the article/supplementary material. Further inquiries can be directed to the corresponding author.

## Author contributions

CT and ZL conceived and designed the experiments; CT and XX conducted the data analysis; CT and ZY performed the experiments; CT wrote the first version of the manuscript. JL and HL contributed to the final version of the manuscript. All authors discussed the result and contributed the final manuscript.

## Funding

This work was supported in part by the science and technology innovation Program of Hunan Province under Grant 2020NK2051 and 2021JJ31158.

## Conflict of interest

The authors declare that the research was conducted in the absence of any commercial or financial relationships that could be construed as a potential conflict of interest.

## Publisher’s note

All claims expressed in this article are solely those of the authors and do not necessarily represent those of their affiliated organizations, or those of the publisher, the editors and the reviewers. Any product that may be evaluated in this article, or claim that may be made by its manufacturer, is not guaranteed or endorsed by the publisher.

## References

[B1] Avitabile V. Camia A. (2018). An assessment of forest biomass maps in Europe using harmonized national statistics and inventory plots. For. Ecol. Manage. 409, 489–498. doi: 10.1016/j.foreco.2017.11.047 29449758 PMC5806600

[B2] Belgiu M. Drăguţ L. (2016). Random forest in remote sensing: A review of applications and future directions. ISPRS J. Photogrammetry Remote Sens. 114, 24–31. doi: 10.1016/j.isprsjprs.2016.01.011

[B3] Biesiada J. Duch W. (2007). Feature selection for high-dimensional data — a Pearson redundancy based filter (Berlin Heidelberg: Springer).

[B4] Breiman L. (2001). Random forests. Mach. Learn. 45 (1), 5–32. doi: 10.1023/A:1010933404324

[B5] Chen W. Xie X. Wang J. Pradhan B. Hong H. Bui D. T.. (2017). A comparative study of logistic model tree, random forest, and classification and regression tree models for spatial prediction of landslide susceptibility. Catena 151, 147–160. doi: 10.1016/j.catena.2016.11.032

[B6] Che S. Tan X. Xiang C. Sun J. Hu X. Zhang X.. (2018). Stand basal area modelling for Chinese fir plantations using an artificial neural network model. J. Forestry Res. 30 (5), 1641–1649. doi: 10.1007/s11676-018-0711-9

[B7] Cooner A. Shao Y. Campbell J. (2016). Detection of urban damage using remote sensing and machine learning algorithms: Revisiting the 2010 Haiti earthquake. Remote Sens. 8 (10), 868. doi: 10.3390/rs8100868

[B8] Cortes C. Vapnik V. (1995). Support-vector networks. Mach. Learn. 20 (3), 273–297. doi: 10.1007/BF00994018

[B9] Duan A. G. Zhang J. G. Tong S. Z. He C. Y. (2013). Polymorphic dominant height and site index models for Chinese fir (Cunninghamia lanceolata) plantations in southern China. Sci. Res. Essays 8 (22), 1010–1021. doi: 10.5897/SRE12.565

[B10] Feng J. Wang J. Yao S. Ding L. (2016). Dynamic assessment of forest resources quality at the provincial level using AHP and cluster analysis. Comput. Electron. Agric. 124, 184–193. doi: 10.1016/j.compag.2016.04.007

[B11] Fujiki S. Okada K.-i. Nishio S. Kitayama K. (2016). Estimation of the stand ages of tropical secondary forests after shifting cultivation based on the combination of WorldView-2 and time-series landsat images. ISPRS J. Photogrammetry Remote Sens. 119, 280–293. doi: 10.1016/j.isprsjprs.2016.06.008

[B12] Gao J. Yuan Q. Li J. Zhang H. Su X. (2020). Cloud removal with fusion of high resolution optical and SAR images using generative adversarial networks. Remote Sens. 12 (1), 191. doi: 10.3390/rs12010191

[B13] Ghasemi N. Tolpekin V. Stein A. (2018). A modified model for estimating tree height from PolInSAR with compensation for temporal decorrelation. Int. J. Appl. Earth Observation Geoinformation 73, 313–322. doi: 10.1016/j.jag.2018.06.022

[B14] Gong Z. Lei W. Chen Z. Gao Y. Zeng S. Zhang G.. (2007). Chinese Soil taxonomy 21, 1.

[B15] Haralick R. M. Shanmugam K. Dinstein I. (1973). Textural features for image classification. IEEE Trans. Systems Man Cybernetics 3 (6), 610–621. doi: 10.1109/TSMC.1973.4309314

[B16] Jin T. T. Fu B. J. Liu G. H. Wang Z. (2011). Hydrologic feasibility of artificial forestation in the semi-arid loess plateau of China. Hydrology Earth System Sci. 15 (8), 2519–2530. doi: 10.5194/hess-15-2519-2011

[B17] Jugran D. Thanruma S. Reddy S. (2005). Forest resource assessment in mohand forest range, uttar pradesh using remote sensing and GIS. J. Indian Soc. Remote Sens. 33 (4), 565–574. doi: 10.1007/bf02990741

[B18] Kahriman A. Günlü A. Karahalil U. (2014). Estimation of crown closure and tree density using landsat TM satellite images in mixed forest stands. J. Indian Soc. Remote Sens. 42 (3), 559–567. doi: 10.1007/s12524-013-0355-3

[B19] Karlson M. Ostwald M. Reese H. Sanou J. Tankoano B. Mattsson E. (2015). Mapping tree canopy cover and aboveground biomass in sudano-sahelian woodlands using landsat 8 and random fores. Remote Sens. 7 (8), 10017–10041. doi: 10.3390/rs70810017

[B20] Kavats O. Khramov D. Sergieieva K. Vasyliev V. (2020). Monitoring of sugarcane harvest in Brazil based on optical and SAR data. Remote Sens. 12 (24):4080. doi: 10.3390/rs12244080

[B21] Lei X. Fu L. LI H. Li Y. Tang S. (2018). Methodology and applications of site quality assessment based on potential mean annual increment. Scientia Silvae Sinicae 054 (012), 116–126. doi: 10.11707/j.1001-7488.20181213

[B22] Li X. Y. Liu Z. H. Wang G. X. Long J. P. Zhang M. (2020). Estimating the growing stem volume of Chinese pine and larch plantations based on fused optical data using an improved variable screening method and stacking algorithm. Remote Sensing 12(5), 101. doi: 10.3390/rs12050871

[B23] Lim K. Treitz P. Wulder M. St-Onge B. Flood M. (2003). LiDAR remote sensing of forest structure. Prog. Phys. Geogr. 27 (1), 88–106. doi: 10.1191/0309133303pp360ra

[B24] Liu J. F. Huang D. N. He Z. Y. Tong S. Z. Yang S. Y. Zhang C. F.. (1982). Preparation and its application of site-index table for Chinese fir. Scientia Silvae Sinicae 18 (3), 266–278.

[B25] Lumbres R. I. C. Seo Y. O. Son Y. M. Doyog N. D. Lee Y. J. J. F. S. Technology (2018). Height-age model and site index curves for acacia mangium and eucalyptus pellita in Indonesia. Forest Sci and Tech 14(1), 1–6. doi: 10.1080/21580103.2018.1452798

[B26] Mirik M. Ansley R. J. Steddom K. Jones D. Rush C. Michels G.. (2013). Remote distinction of a noxious weed (Musk thistle: CarduusNutans) using airborne hyperspectral imagery and the support vector machine classifier. Remote Sens. 5 (2), 612–630. doi: 10.3390/rs5020612

[B27] Mountrakis G. Im J. Ogole C. (2011). Support vector machines in remote sensing: A review. ISPRS J. Photogrammetry Remote Sens. 66 (3), 247–259. doi: 10.1016/j.isprsjprs.2010.11.001

[B28] Ou Q. Lei X. Shen C. (2019). Individual tree diameter growth models of larch–Spruce–Fir mixed forests based on machine learning algorithms. Forests, 10(2), 187. doi: 10.3390/f10020187

[B29] Pu R. Cheng J. (2015). Mapping forest leaf area index using reflectance and textural information derived from WorldView-2 imagery in a mixed natural forest area in Florida, US. Int. J. Appl. Earth Observation Geoinformation 42, 11–23. doi: 10.1016/j.jag.2015.05.004

[B30] Pueschel P. Buddenbaum H. Hill J. (2012). An efficient approach to standardizing the processing of hemispherical images for the estimation of forest structural attributes. Agric. For. Meteorology 160, 1–13. doi: 10.1016/j.agrformet.2012.02.007

[B31] Reich P. B. (2012). Key canopy traits drive forest productivity. Proc. R. Soc. B: Biol. Sci. 279 (1736), 2128–2134. doi: 10.1098/rspb.2011.227 PMC3321697 22279168

[B32] Rodriguez-Galiano V. Sanchez-Castillo M. Chica-Olmo M. Chica-Rivas M. (2015). Machine learning predictive models for mineral prospectivity: An evaluation of neural networks, random forest, regression trees and support vector machines. Ore Geology Rev. 71, 804–818. doi: 10.1016/j.oregeorev.2015.01.001

[B33] Shao Y. Lunetta R. S. (2012). Comparison of support vector machine, neural network, and CART algorithms for the land-cover classification using limited training data points. ISPRS J. Photogrammetry Remote Sens. 70, 78–87. doi: 10.1016/j.isprsjprs.2012.04.001

[B34] Shataee S. Kalbi S. Fallah A. Pelz D. (2012). Forest attribute imputation using machine-learning methods and ASTER data: comparison of k-NN, SVR and random forest regression algorithms. Int. J. Remote Sens. 33 (19), 6254–6280. doi: 10.1080/01431161.2012.682661

[B35] Silva Guimarães U. de Lourdes Bueno Trindade Galo M. da Silva Narvaes I. de Queiroz da Silva A. (2020). Cosmo-SkyMed and TerraSAR-X datasets for geomorphological mapping in the eastern of marajó island, Amazon coast. Geomorphology 350:106934. doi: 10.1016/j.geomorph.2019.106934

[B36] Venkatalaxmi A. Padmavathi B. S. Amaranath T. (2004). A general solution of unsteady stokes equations. Fluid Dynamics Res. 35 (3), 229–236. doi: 10.1016/j.fluiddyn.2004.06.001

[B37] Verrelst J. Muñoz J. Alonso L. Delegido J. Rivera J. P. Camps-Valls G.. (2012). Machine learning regression algorithms for biophysical parameter retrieval: Opportunities for sentinel-2 and -3. Remote Sens. Environ. 118, 127–139. doi: 10.1016/j.rse.2011.11.002

[B38] Wang L. Dai Y. Sun J. Wan X. (2017). Differential hydric deficit responses of robinia pseudoacacia and platycladus orientalis in pure and mixed stands in northern China and the species interactions under drought. Trees 31 (6), 2011–2021. doi: 10.1007/s00468-017-1605-8

[B39] Wang B. Waters C. Orgill S. Gray J. Cowie A. Clark A.. (2018). High resolution mapping of soil organic carbon stocks using remote sensing variables in the semi-arid rangelands of eastern Australia. Sci. Total Environ. 630, 367–378. doi: 10.1016/j.scitotenv.2018.02.204 29482145

[B40] Wolter P. T. Townsend P. A. Sturtevant B. R. (2009). Estimation of forest structural parameters using 5 and 10 meter SPOT-5 satellite data. Remote Sens. Environ. 113 (9), 2019–2036. doi: 10.1016/j.rse.2009.05.009

[B41] Xu Y. Smith S. E. Grunwald S. Abd-Elrahman A. Wani S. P. (2018). Effects of image pansharpening on soil total nitrogen prediction models in south India. Geoderma 320, 52–66. doi: 10.1016/j.geoderma.2018.01.017

[B42] Yalew S. G. van Griensven A. Mul M. L. van der Zaag P. (2016). Land suitability analysis for agriculture in the abbay basin using remote sensing, GIS and AHP techniques. Modeling Earth Syst. Environ. 2 (2), 101. doi: 10.1007/s40808-016-0167-x

[B43] Yang X. Yu Y. Li M. (2018). Estimating soil moisture content using laboratory spectral data. J. Forestry Res. 30 (3), 1073–1080. doi: 10.1007/s11676-018-0633-6

[B44] Yu Y. Chen J. M. Yang X. Fan W. Li M. He L. (2017a). Influence of site index on the relationship between forest net primary productivity and stand age. PLoS One 12 (5), e0177084. doi: 10.1371/journal.pone.0177084 28493995 PMC5426654

[B45] Yu Y. Li M. Fu Y. (2017b). Forest type identification by random forest classification combined with SPOT and multitemporal SAR data. J. Forestry Res. 29 (5), 1407–1414. doi: 10.1007/s11676-017-0530-4

[B46] Yu X. Zhang M. Yang H. Chen C. (2019). An NFI-based site quality evaluation of Chinese fir plantation. J. Sustain. Forestry 39 (2), 137–152. doi: 10.1080/10549811.2019.1623051

[B47] Zhang C. Denka S. Cooper H. Mishra D. R. (2018). Quantification of sawgrass marsh aboveground biomass in the coastal Everglades using object-based ensemble analysis and landsat data. Remote Sens. Environ. 204s 366–379. doi: 10.1016/j.rse.2017.10.018

[B48] Zhang J. Su Y. Wu J. Liang H. (2015). GIS based land suitability assessment for tobacco production using AHP and fuzzy set in Shandong province of China. Comput. Electron. Agric. 114, 202–211. doi: 10.1016/j.compag.2015.04.004

[B49] Zhao Q. Yu S. Zhao F. Tian L. Zhao Z. (2019). Comparison of machine learning algorithms for forest parameter estimations and application for forest quality assessments. For. Ecol. Manage. 434, 224–234. doi: 10.1016/j.foreco.2018.12.019

[B50] Zhou J. J. Zhao Z. Zhao J. Zhao Q. Wang F. Wang H. (2013). A comparison of three methods for estimating the LAI of black locust (Robinia pseudoacacia l.) plantations on the loess plateau, China. Int. J. Remote Sens. 35 (1), 171–188. doi: 10.1080/01431161.2013.866289

